# Method validation for determination of nine pesticides in okra and their mitigation using different solutions

**DOI:** 10.1371/journal.pone.0260851

**Published:** 2021-12-02

**Authors:** Anjana Srivastava, Gajan Pal Singh, Prakash Chandra Srivastava

**Affiliations:** 1 Department of Chemistry, College of Basic Sciences and Humanities, Govind Ballabh Pant University of Agriculture and Technology, Pantnagar, US Nagar, Uttarakhand, India; 2 Department of Soil Science, College of Agriculture, Govind Ballabh Pant University of Agriculture and Technology, Pantnagar, US Nagar, Uttarakhand, India; South China Agricultural University, CHINA

## Abstract

In this paper we optimized QuEChERS method for extraction of nine pesticides viz. acephate, acetamiprid, chlorpyrifos, cypermethrin, imidacloprid, thiamethoxam, profenofos (insecticides), carbendazim and tebuconazole (fungicides) and performed their quantitative estimation in okra crop by HPLC-UV and GC-ECD. Decontamination treatments namely washing with running tap water, soaking in lukewarm water (50–60°C), soaking in solutions of 1% NaCl, 5% NaHCO_3_, 2% CH_3_COOH, 0.01% KMnO_4_ and three commercial formulations were also done for ten minutes every time, to calculate the extent of pesticide removal from okra. Results revealed that the proposed extraction method was efficient, inexpensive, accurate, rapid and precise and can suitably be used for the simultaneous quantitative determination of the above pesticides. The standard curve was linear over the concentration range of 0.05–5μg g^-1^ with R^2^ close to one (0.999). Soaking of okra in 2% acetic acid and then washing proved as the best decontamination treatments for all the pesticides. It showed the highest relative decontaminating capacity in comparison to the other solutions tested. Since the pesticide residues are usually present in higher amount in vegetables being consumed, it is of utmost importance to keep an eye over the use of pesticides to protect the crops.

## Introduction

Vegetables are an essential component of human diet and are consumed in all forms i.e. raw or cooked and peeled or unpeeled form as they are a good source of vitamins that are essential for humans [1]. Okra [*Abelmoschus esculentus* (L.) Moench] commonly known as lady finger is an important vegetable crop of India, belonging toMalvaceae family. It is cultivated during spring-summer and rainy seasons but at fruiting stage, it is heavily attacked by shoot and fruit borers [2]. In order to control these pests in okra as well as other vegetable crops different insecticides (organophosphate, synthetic pyrethroid and neonicotinoid groups) and fungicides (benzimidazole and triazole groups) are applied. Pesticides having low mammalian toxicity and less risk of contamination are recommended [3]. In spite of several IPM recommendations there is an indiscriminate use of pesticides by vegetable growers which often leads to the build-up of pesticide residues in vegetable crops. Most often pre-harvest intervals (PHI) are also not observed by the farmers which result in considerable quantities of pesticides that are absorbed by vegetables. The consumption of pesticides above their maximum residue limits (MRL) or acceptable daily intake (ADI) can lead to several harmful effects. Hence the monitoring and decontamination of pesticides are extremely important aspects for making the vegetables safe for consumption. Food safety is a major concern worldwide and in a country like India where a big percentage of population is below poverty line it becomes all the more important that such simple and effective strategies may be devised which can be adopted by the common man to effectively reduce pesticide residues load from vegetables. Reports on pesticidal contamination of vegetables has been published by several researchers [4–6]. Researchers throughout the world are actively engaged in investigating newer techniques for efficacious removal of pesticide residues. These can range from the use of household chemicals as decontaminants or to more recent non-thermal processes [7].

Multi-residue analysis procedures are frequently being used for extraction and estimation of different groups of pesticides from the samples due to their advantages over conventional methods [8, 9]. In the present study, validation of modified multi-residue QuEChERs methods for the extraction of pesticides from okra and quantification through HPLC-UV and GC-ECD was done. Method validation included suitability, specificity and selectivity, precision, accuracy, linearity, robustness, limit of detection, and limit of quantification. The efficacy of different decontaminants which can mitigate the pesticide levels was also compared so that the pesticide residues remain within the recommended maximum residue limits (MRL) or acceptable daily intake (ADI) limits at the time of consumption.

## Materials and methods

The analytical grade standards of acephate, acetamiprid, carbendazim chlorpyrifos, cypermethrin, imidacloprid, tebuconazole and thiamethoxam were procured from Sigma Aldrich, India and profenofos was provided as CRM (Certified Reference Material) by IARI, New Delhi. The formulations of the above-mentioned pesticides were procured from the local market.

### Experimental details

The experiment was conducted during March 2021 to June 2021. Okra crop (cv. Parbhani kranti) was planted in Vegetable Research Centre (CRC) Pantnagar, in 5m x 5m plots with plant to plant spacing of 60cm x 45cm. The pesticides used for spray on okra were the mixture of pesticides formulations at the recommended dosages depicted in Table 1.

**Table 1:** Pesticide formulations and applied doses in okra crop.

Pesticide	Formulation	Dose applied (g a.i./ha)
Acephate	Acemain 75%SC	1500g a.i/ha
Acetamiprid	Ennova 20%SP	100g a.i/ha
Carbendazim	Zen 50%WP	250g a.i/ha
Chlorpyrifos	Tricel 20%EC	400g a.i/ha
Cypermethrin	Cyperguard 10%EC	100g a.i/ha
Imidacloprid	Imidaveer 17.8%SL	50g a.i/ha
Profenofos	Celcron 50%EC	50g a.i/ha
Tebuconazole	Folicur 25.9%EC	300g a.i/ha
Thiamethoxam	Ultra 25%WG	100g a.i/ha

About 250–300 g of the okra crop was plucked at 50–60% fruiting stage for the recovery studies before spraying and thereafter the crop was sprayed with the pesticide formulation mixture for only once at the recommended doses. The samples of okra (1.5–2.0 kg) were collected after 48 hrs of spraying of pesticide mixture formulation and brought to the Agrochemical laboratory, Department of Chemistry, College of Basic Sciences and Humanities, Pantnagar, for performing extraction, residue analysis and decontamination of pesticides.

### Linearity curve, LOD and LOQ

A standard 100 μg g^-1^ stock solution of acephate, carbendazim, acetamiprid, thiamethoxam, imidacloprid and tebuconazole pesticides were prepared individually in acetonitrile. The stock solutions of above pesticides were diluted to 5 μg g^-1^ and thereafter serial dilutions of 0.05, 0.1, 1.0, 2.0, 3.0, 4.0 μg g^-1^ were made by further dilution with acetonitrile of HPLC grade. Peak area for each concentration was determined through HPLC-UV analysis and calibration curves were plotted. Similarly, standard stock solutions of 100 μg g^-1^ of chlorpyrifos, cypermethrin and profenofos were prepared in hexane and were diluted to 5 μg g^-1^. Serial dilutions of varying concentrations (0.05, 0.1, 1.0, 2.0, 3.0, 4.0 μg g^-1^)were done by further diluting with n- hexane and calibration curves were plotted after determining their peak area by GC-ECD.

The LOD and LOQ values were also determined using the mathematical equations

LOD = 3.3 x σ/S and LOQ = 10 x σ/S

Where σ = Standard deviation of the intercept and S = Slope of calibration curve.

Concentration of the pesticides were determined by using calibration curve and regression equation of the linearity graph.

### Accuracy

In order to establish the accuracy of analytical methods adopted and to know the efficiency of extraction and clean up steps’ recovery studies were undertaken by spiking the okra samples with known amounts of reference standards. The okra samples were fortified at 0.05, 0.10 and 1.0 μg g^-1^ for all the pesticides viz. acephate, acetamiprid, carbendazim chlorpyrifos, cypermethrin, imidacloprid, thiamethoxam, profenofos and tebuconazole.

### Precision

Repeatability tests were performed by analyzing three replicate samples of all the pesticides at 100% test concentrations. The precision of the method was established throughinterday and intraday precision studies by measuring the response of all the pesticides three times a day and on three different days.

### Robustness

The robustness of the methods was confirmed by analyzing the pesticide samples with deliberate variations in the optimized experimental conditions. The changes in the responses of pesticides were noted and the results were calculated in terms of% RSD. For HPLC method, robustness of the method was determined by making deliberate changes in flow rate (±0.5 mL min^-1^) and λ max (±2 nm) using concentration of 0.1 μg g^-1^ for all the pesticides. For GC method, robustness was measured by checking for variations on the carrier gas flow rate, ±10°C on the initial oven temperature and ±50% on the split ratio. For each set of variation, three replicate injections of the standard solution were performed.

### Extraction of pesticides from okra

The extraction of all the pesticides was done using standard QuEChERS method [10] with slight modifications like reduction of sample size, no use of buffering agents and graphitized carbon black etc. which are commonly used in typical QuEChERS method. A representative 5 g sample of okra was taken in six 50 ml centrifuge tube (2 sets of three). To one set 10 mL distilled water and 10 mL of n-Hexane were added whereas in the other set 10 mL distilled water and 10 mL of acetonitrile was added. The contents of both the sets were vortexed for two minutes and the mixtures were allowed to stand of 10 minutes. Thereafter, 3g of anhydrous MgSO_4_ and 1 g of NaCl were added to the mixtures and werere-vortexed for 2 minutes followed by centrifugation for 5 minutes at 4000 rpm. After this, 1g MgSO_4_ and 150 mg PSA (primary secondary amine) reagent were added to the aliquot and the mixture was further centrifuged at 4000 rpm for 5 minutes. Phase separation took place after which the upper organic layer was taken and passed through solid phase extraction (SPE) cartridge for further cleanup. It was then filtered through 0.45 μm Poly tetra fluoro ethylene (PTFE) disc filter for analysis by GC-ECD / HPLC-UV. The developed modified method, demonstrated acceptable accuracy and precision with higher recoveries of all the nine pesticides and no matrix effect interference, indicating its higher effectiveness.

### Optimization of chromatographic conditions

The quantitative determination of acephate, acetamiprid, carbendazim, imidacloprid, thiamethoxam and tebuconazole pesticides was done by HPLC using Dionex Ultimate 3000 system equipped with RP-C18 column (250x4.6mm) (particle size-5 μm), injector loop of 20μl, UV-VIS detector and dual pump. The HPLC conditions (mobile phase, wavelength and flow rate retention times (t_r_)) during the analysis were different for individual or a combo of pesticides (Table 2).

**Table 2:** Optimised conditions for pesticides analysed by HPLC–UV.

Pesticide	Mobile phase (v/v)	Wavelength (nm)	Flow rate ml min^-1^	Retention time (t_r_) in min.
Acephate and it’s metabolite methamidophos	Acetonitrile/water (90:10)	195	1.0	2.5
Imidacloprid	Methanol/water (45:55)	254	0.5	12.3
Acetamiprid				15.8
Thiamethoxam	Acetonitrile/water (45:55)	254	0.5	7.0
Carbendazim				8.0
Tebuconazole	Acetonitrile/water (0.1% formic acid) (70:30)	240	1.0	5.0

The analysis of chlorpyrifos, cypermethrin and profenofos was done by GC (Thermofisher Scientific, Trace 1110) mounted with ECD and a capillary column (30 m x 0.25 mm i.d. having a film thickness of 0.25μm). Optimization of GC conditions was done by varying the column temperatures, gas flow rate etc. An initial column temperature of 100°C—increase @ 25°C min^-1^ for 5 min. up to 180°C–increase @ 5°C min^-1^ for 20 min. up to 280°C followed by a final ramp rate of 10°C min^-1^ to reach to a temperature of 300°C was finalized for the pesticides analysis. The injection volume was 1 μl and the injector and detector temperatures were 250° and 300°C, respectively. Nitrogen (99.99% purity) was used as the carrier gas at a flow rate of 1.2 ml min^-1^ and the total run time was taken as 45min. for the elution of the three pesticides. Under the abovementioned GC-ECD conditions the peaks of the pesticides were well resolved and retention times of chlorpyrifos, profenofos and cypermethrin were 17.2, 19.9 and 29.9min. respectively.

### Decontamination treatments on okra

About 4–5 kg of okra, after 48 h of pesticides formulation mixture spray, was brought from the agricultural field and was divided in two parts for analysis. The samples of okra from both the parts, were imperilled to nine different decontamination treatments viz., washing with running tap water, soaking in lukewarm water (50–60°C), soaking in 1% NaCl, soaking in 5% NaHCO_3_ aqueous solution, soaking in 2% CH_3_COOH, soaking in 0.01% KMnO_4_ and dipping in commercially available decontaminant formulations (Veggi clean, Nimwash and Arka herbiwash) for 10 min. every time before pesticides extraction. One sample was analysed as such i.e. without being subjected to any decontamination treatment and was termed as control. The extraction of pesticides both for HPLC and GC analysis was done as described in the preceding section.

### Statistical analysis

Standard deviation (SD) and Relative Standard deviation (RSD) were calculated for determining the reliability of data. The decontamination of pesticides was expressed as mean percent removal. The data on decontamination studies were analysed in randomized block design (RBD) set up and test of significance was carried out by F-test. The critical difference (CD) values were computed at p≤0.05.

## Results and discussion

### Method validation

The method was validated for various parameters such as precision, linearity, accuracy, limit of detection and quantification on the basis of recovery experiments (Table 3). Linear correlations were obtained between absorbance and concentration for different pesticides in the range of 1.0–5.0 μg g^-1^ for all the pesticides. The recovery of different pesticides ranged between 82.0–86.7, 82.7–87.3 and 85.192.0 when the okra samples were fortified at 0.05, 0.1 and 1.0 μg g^-1^ rates by pesticide mixtures respectively. The relative standard deviation (RSD) values were < 5%. The tested validation parameters were found to be within acceptable limits.

**Table 3:** Method validation data and MRLs of pesticides in okra.

Pesticides	Linearity (R^2^)	LOD (μg g^-1^)	LOQ (μg g^-1^)	Recovery (%) with ± RSD (μg g^-1^)			MRL (EU) (μg g^-1^)
				0.05	0.1	1.0	
**Acephate and it’s metabolite methamidophos**	0.999	0.01	0.05	82.0 ±4.0	82.7±3.1	85.1 ±1.5	
**Imidacloprid**	0.999	0.01	0.05	86.0 ±3.5	86.0±3.6	91.8 ±3.7	0.5
**Acetamiprid**	0.999	0.01	0.05	86.0± 4.0	86.3±1.5	88.6±3.0	0.5
**Thiamethoxam**	0.999	0.01	0.05	86.7±2.3	87.3±1.1	89.7±2.6	0.3
**Carbendazim**	0.998	0.01	0.05	82.0±3.5	86.3±2.1	87.6±1.2	0.1
**Tebuconazole**	0.989	0.01	0.05	85.9±4.9	87.1±2.2	92.0±2.2	0.02
**Chlorpyrifos**	0.999	0.005	0.015	80.0±2.0	87.7±1.5	91.5±4.8	0.3
**Profenofos**	0.995	0.005	0.015	86.7±2.3	87.3±2.1	90.8±1.0	0.01
**Cypermethrin**	0.989	0.005	0.015	88.7±3.1	89.3±2.5	90.1±1.7	0.05

### Optimization of chromatographic conditions

Several trials were done for optimizing the mobile phases, λ max and flow rate determinations for different pesticides for HPLC analysis. Finally, acetonitrile: water and methanol: water, in different proportions, in which the peaks were well resolved with clear baseline separation, were selected. In case of GC the obtained results after optimization revealed that studied variations of GC conditions do not cause any significant changes in system suitability. On this basis the methods can be considered robust.

### Decontamination studies

**Fig 1:**
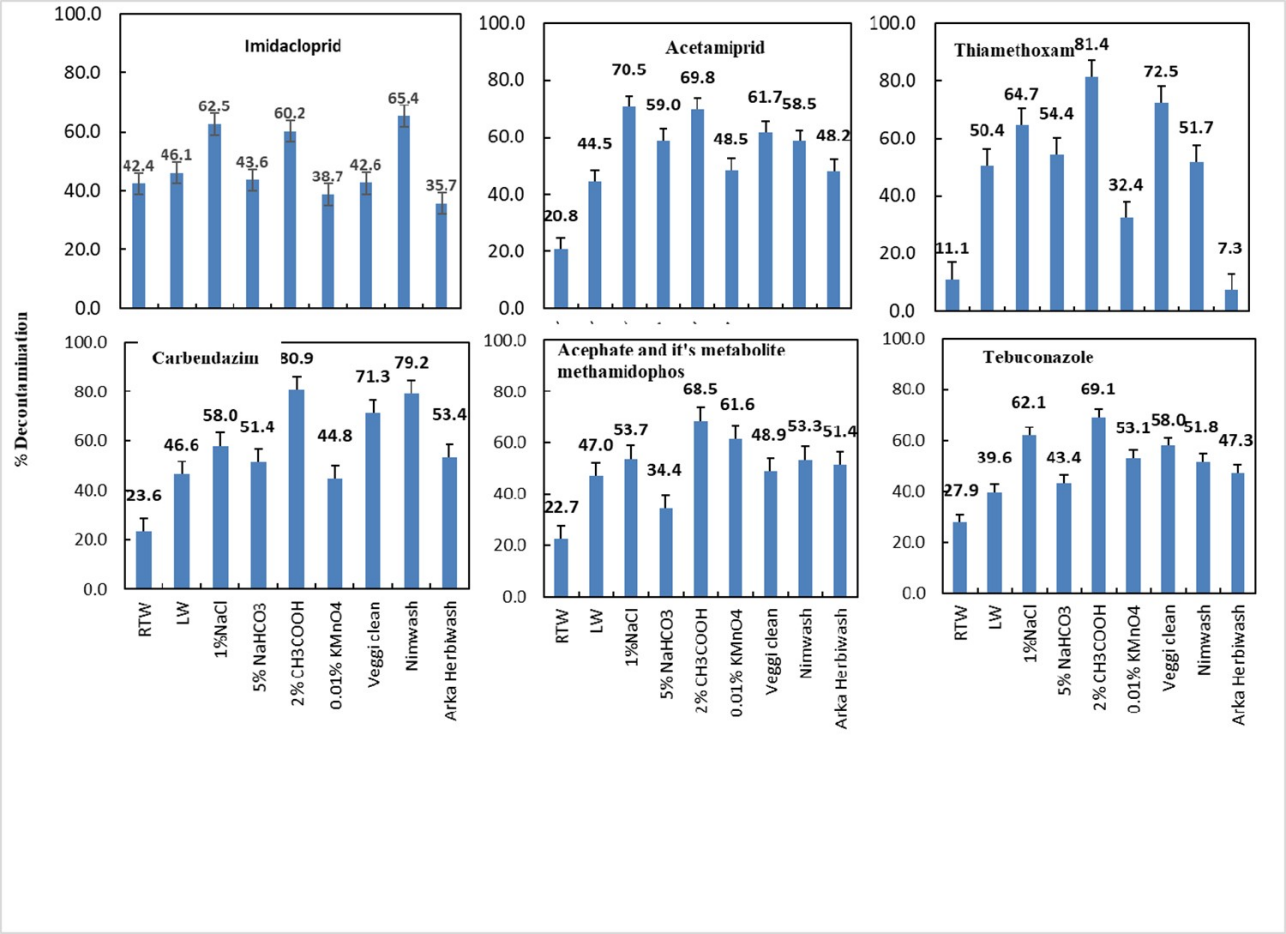
Effect of different decontamination treatments on % removal of pesticides analyzed by HPLC-UV on okra. The vertical bars indicate critical difference at P ≤ 0. The numerical values against histograms indicate percent (%) decontamination of pesticide residues.

**Fig 2:**
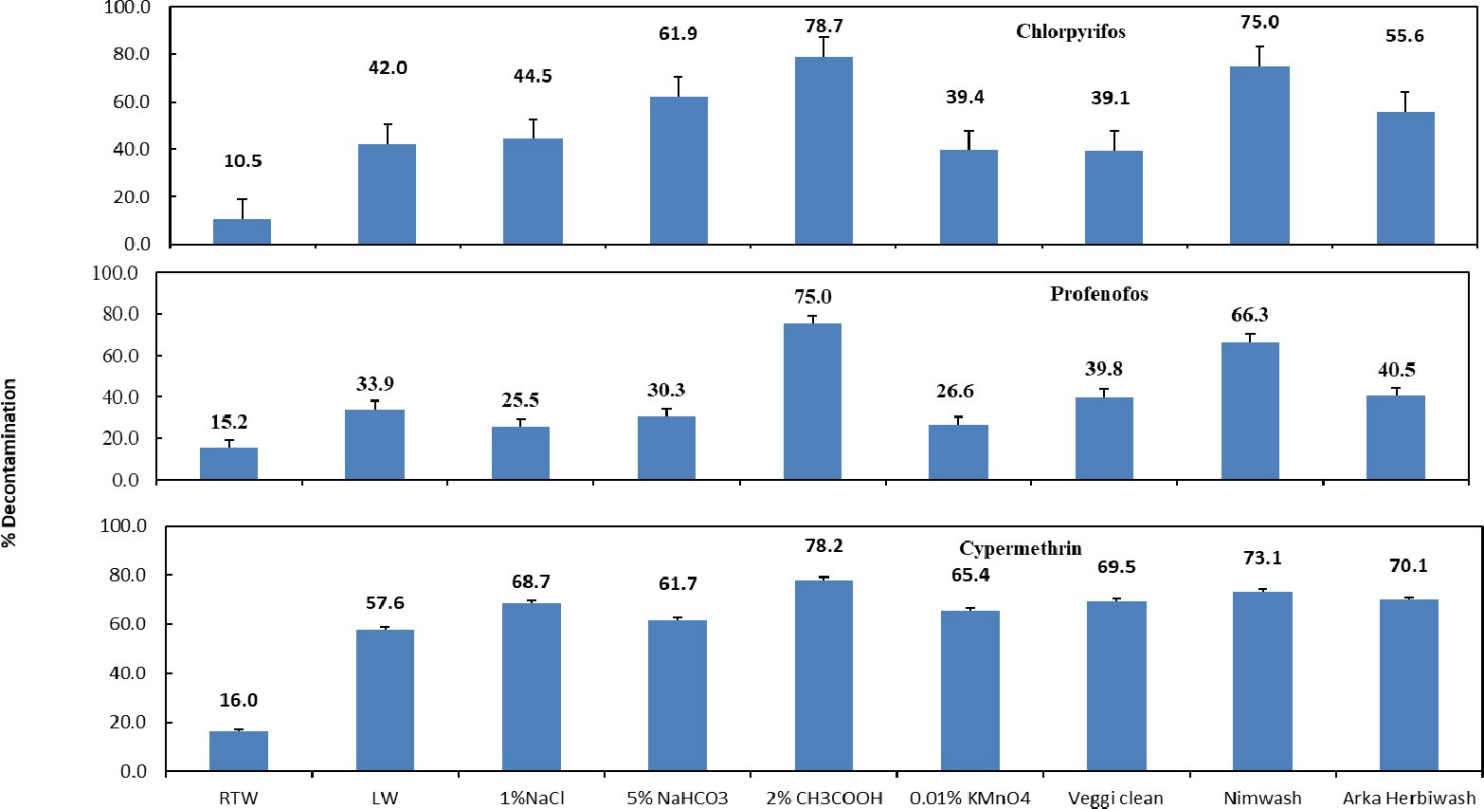
Effect of different decontamination treatments on % removal of pesticides analyzed by GC-ECD on okra. The vertical bars indicate critical difference at P ≤ 0.05. The numerical values against histograms indicate percent (%) decontamination of pesticide residues.

Data related to the effect of different decontaminants on pesticides residues mitigation in okra are presented in Table 4. It is evident from the table that the initial deposits of all the pesticides in control were the highest. A 2% solution of CH_3_COOH served to be the best decontaminant solution for all the above pesticides (insecticides and fungicides) as it could remove more than 50% of all the pesticide residues, though some other reagents like soaking in 1% NaCl and commercial decontaminants were also effective in pesticide removal. It has been reported that adding reagents like CH_3_COOH, NaCl, H_2_O_2_ etc. to the washing water can lead to a high degree of decrease in the pesticide residues as it affects the chemical bonds between pesticide and crop surface [11]. Srivastava et al. [12], have also reported that brine solutions of 1 and 5% concentrations were effective in decontaminating pesticide residues from okra. Washing with lukewarm or hot water was also found to be effective in pesticide mitigation as the physical and chemical characteristics of the pesticides too, determine their disappearance and removal rate. It has been reported in previous studies that boiling of vegetables was more effective in the removal of pesticide residues than washing [13, 14]. Washing solutions effectively reduce pesticide residues owing to the chemical properties of these solutions: acidity, alkalinity, presence of electrolytes, and surfactants etc. The plant cuticle in fruits and vegetables can be regarded as an electrically asymmetric membrane in which a clear electrokinetic gradient is likely to be established across cuticle [15, 16]. Thus, the outer surface remains uncharged, the inner surface supports a net negative charge due to a Donnan-like membrane potential associated with polysaccharides present in the inner side of the crop cuticle [15]. In our studies we found that solutions of 5% NaHCO_3_ and 0.01% KMnO_4_ were only slightly effective in dislodging pesticides residues from okra though it has been demonstrated that the use of sodium bicarbonate, commonly known as baking soda (10 mg mL^-1^), was effective for the removal of some pesticide residues like thiabendazole and phosmet from apples [17]. The commercial decontaminants like Nimwash and Veggie Clean too, were effective in lowering of pesticide residues on okra crop. However, the residues that were found even after water and salt water washing, may be due to lower water solubility of pesticides or due to strong bonding between the insecticide molecules and waxy layer of crop. The removal extent of pesticides by the use of different solutions is depicted in Figs 1 and 2. As evident from the figures, the maximum removal percent is of carbendazim followed by cypermethrin and neonicotinoids especially thiamethoxam and acetamiprid with all the decontaminants. Among organaphosphates, acephate and it’s metabolite methamidophos and to some extent chlorpyrifos could be dislodged by different decontamination treatments.

**Table 4:** Residues of pesticides in okra after different decontamination treatments.

Decontamination Treatments	Residues (mg/kg)								
	Acephate and it’s metabolite methamidophos	Cypermethrin	Acetamiprid	Imidacloprid	Profenofos	Chlorpyrifos	Thiamethoxam	Tebuconazole	Carbendazim
**Control (No washing treatment)**	4.298	0.101	0.151	0.186	0.382	0.040	0.277	9.541	1.807
**Washing with Running Tap Water**	3.324	0.084	0.119	0.121	0.324	0.036	0.246	6.877	1.381
**Washing with Lukewarm water**	2.278	0.043	0.084	0.103	0.253	0.023	0.137	5.760	0.965
**1% NaCl**	1.989	0.031	0.044	0.074	0.285	0.022	0.098	3.620	0.759
**5% NaHCO**_**3**_	2.818	0.038	0.062	0.106	0.267	0.015	0.126	5.402	0.878
**2% acetic acid**	1.353	0.022	0.045	0.070	0.096	0.009	0.052	2.951	0.345
**0.01% KMnO**_**4**_	1.651	0.035	0.078	0.116	0.281	0.024	0.187	4.472	0.998
**Veggie Clean**	2.195	0.031	0.058	0.115	0.230	0.024	0.076	4.005	0.519
**Nimwash**	2.005	0.027	0.063	0.071	0.129	0.010	0.134	4.598	0.376
**ArkaHerbiwash**	2.089	0.030	0.078	0.113	0.227	0.018	0.257	5.027	0.842
**SEm (±)**	0.077	0.003	0.002	0.007	0.005	0.001	0.006	0.099	0.030
**CD (p≤0.05)**	0.229	0.001	0.007	0.022	0.015	0.004	0.019	0.293	0.090

### Relationship between MRL and decontamination extent of different pesticides

The maximum residue level (MRL) is the index set to represent the permitted level of pesticide residues, and it indicates, that a produce containing residues below the MRL value is safe to consume [7]. The reactivity of different pesticides with solvents mainly depends upon the chemical characteristics of pesticide molecules and nature of the vegetable and fruit [18]. The decontamination effect is dependent on the systemic and contact nature of pesticides. Systemic pesticides can penetrate into the flesh of vegetables, whereas contact pesticides mostly remain on the surface. It is evident from Table 4 that the highest concentrations of detected pesticides were recorded for tebuconazole (fungicide) followed by acephate and it’s metabolite methamidophos, carbendazim, thiamethoxam, profenofos, imidacloprid, acetamiprid, cypermethrin and chlorpyrifos. The results were assessed according to the MRLs provided by European regulations for each pesticide in okra. None of the decontamination treatments could bring down the residue levels of systemic pesticides like acephate and it’s metabolite methamidophos, carbendazim and tebuconazole below the recommended MRL values as depicted in Table 2. Chlorpyrifos, being a contact insecticide, could be easily brought down below it’s recommended MRL (0.3μg g^-1^) after all the treatments but profenofos (contact insecticide) could not be removed. This might be due to the reason that fruit and vegetables have pores in which the pesticide molecules can get trapped or absorbed making them difficult to be rinsed off once they have been applied. Inspite of being systemic in nature, the MRLs of neonicotinoids (acetamiprid, imidacloprid and thiamethoxam) are higher in comparison to other group of pesticides as they are considered to be less toxic [19] and thus after decontamination treatments their residues which were below 0.5 μg g^-1^ fell between their recommended MRL values (0.3 to 0.5 μg g^-1^) Pyrethroids are non- polar compounds with low persistence and a tendency to hydrolyze in acidic and alkaline medium. Cypermethrin, a type II pyrethroid, could not be decontaminated much by simple tap water washing probably due to its apolar nature but other treatments could bring down the insecticide residues below the MRL values i.e.< 0.05μg g^-1^.

## Conclusions

As per the results obtained, recovery percentages of pesticides were between 82 to 92 percent with RSD < 5%, which indicates that the QuEChERS method followed with modification and optimization of chromatographic conditions was suitable for extraction and estimation of pesticides by HPLC-UV and GC-ECD. The observed high levels of pesticide residues may represent a potential health risk for consumers as several vegetables are consumed raw. Household processing, including washing, peeling, and cooking, and treatment with mild chemical solutions is necessary to reduce the amount of pesticide residues in vegetables. The pesticide residues in all the crops should be regularly monitored in order to better protect consumer’s health. The combination of chemical solutions with modern decontamination technologies should be tried, which can result in ensuring safe supply of fresh fruits and vegetables to consumers.
